# Cancer-associated fibroblasts enact field cancerization by promoting extratumoral oxidative stress

**DOI:** 10.1038/cddis.2016.492

**Published:** 2017-01-19

**Authors:** Jeremy Soon Kiat Chan, Ming Jie Tan, Ming Keat Sng, Ziqiang Teo, Terri Phua, Chee Chong Choo, Liang LI, Pengcheng Zhu, Nguan Soon Tan

**Affiliations:** 1 School of Biological Sciences, Nanyang Technological University, 60 Nanyang Drive, Singapore; 2 Department of Microbiology, Tumor and Cell Biology, Karolinska Institutet, Nobelsväg 16, StockholmSweden; 3 Lee Kong Chian School of Medicine, Nanyang Technological University, 50 Nanyang Avenue, Singapore; 4 Institute of Molecular and Cell Biology, A*STAR, 61 Biopolis Drive, Proteos, Singapore; 5 KK Women's and Children Hospital, 100 Bukit Timah Road, Singapore

## Abstract

Histological inspection of visually normal tissue adjacent to neoplastic lesions often reveals multiple foci of cellular abnormalities. This suggests the presence of a regional carcinogenic signal that spreads oncogenic transformation and field cancerization. We observed an abundance of mutagenic reactive oxygen species in the stroma of cryosectioned patient tumor biopsies, indicative of extratumoral oxidative stress. Diffusible hydrogen peroxide (H_2_O_2_) was elevated in the conditioned medium of cultured skin epithelia at various stages of oncogenic transformation, and H_2_O_2_ production increased with greater tumor-forming and metastatic capacity of the studied cell lines. Explanted cancer-associated fibroblasts (CAFs) also had higher levels of H_2_O_2_ secretion compared with normal fibroblasts (FIBs). These results suggest that extracellular H_2_O_2_ acts as a field effect carcinogen. Indeed, H_2_O_2_-treated keratinocytes displayed decreased phosphatase and tensin homolog (PTEN) and increased Src activities because of oxidative modification. Furthermore, treating FIBs with CAF-conditioned medium or exogenous H_2_O_2_ resulted in the acquisition of an oxidative, CAF-like state. *In vivo*, the proliferative potential and invasiveness of composite tumor xenografts comprising cancerous or non-tumor-forming epithelia with CAFs and FIBs could be attenuated by the presence of catalase. Importantly, we showed that oxidatively transformed FIBs isolated from composite tumor xenografts retained their ability to promote tumor growth and aggressiveness when adoptively transferred into new xenografts. Higher H_2_O_2_ production by CAFs was contingent on impaired TGF*β* signaling leading to the suppression of the antioxidant enzyme glutathione peroxidase 1 (GPX1). Finally, we detected a reduction in Smad3, TAK1 and TGF*β*RII expression in a cohort of 197 clinical squamous cell carcinoma (SCC) CAFs, suggesting that impaired stromal TGF*β* signaling may be a clinical feature of SCC. Our study indicated that CAFs and cancer cells engage redox signaling circuitries and mitogenic signaling to reinforce their reciprocal relationship, suggesting that future anticancer approaches should simultaneously target ligand receptor and redox-mediated pathways.

## 

Tumor initiation, growth and propagation are frequently accompanied by desmoplasia and the acquisition of a reactive stroma mainly comprising cancer-associated fibroblasts (CAFs).^1, 2, 3^ The dynamic and reciprocal relationship between the epithelial and mesenchymal compartments of the tumor dictates almost every aspect of cancer progression, even governing the efficacy of therapy and influencing the risk of disease relapse.^4, 5^ Importantly, a population of epithelial and stromal cells adjacent to the primary neoplastic lesion acquires early genetic changes but lacks histopathology, indicating that tumors prime the surrounding tissue for malignancy, an effect known as field cancerization.^6, 7^ Field cancerization results in multifocal primary tumors in close proximity with a higher chance of recurrence after surgical resection of malignant tumors.^8^ Although genetic and epigenetic anomalies have been well described as the driving force behind field cancerization of premalignant epithelia,^6^ mediators of field cancerization by the stroma remain poorly understood.

The expansion of the cancer field requires soluble factors that promote oncogenic transformation in a non-cell-autonomous manner and prime the adjacent naïve stroma to support tumor growth. Although these functions are usually ascribed to growth factors and cytokines, reactive oxygen species (ROS), which are persistently elevated in almost all cancers, have been recently identified as critical intermediates of cellular signaling.^9, 10, 11^ The oxidative modification of proteins by ROS modulates intracellular signaling to influence cellular behavior and contribute to the pathophysiology of many human diseases.^12, 13^ ROS are by-products of aberrant metabolism in the tumor epithelia, and their cell-autonomous mutagenic effects have been studied extensively. Much less is known about the regulation of ROS in CAFs and how ROS impact field cancerization. Notably, not all ROS are well suited for paracrine cell signaling. Superoxide anions (O2-) and hydroxyl radicals (OH•) have a very short diffusion distance because they are highly reactive, resulting in a very short half-life in the aqueous phase.^14^ They also have a very low permeability coefficient for lipid bilayers.^15^ In contrast, hydrogen peroxide (H_2_O_2_) has a longer half-life, is lipid soluble and can diffuse across the cellular plasma membrane, making it an ideal field effect carcinogen. In this study, we investigated the regulation of ROS, and in particular, H_2_O_2_ production, in CAFs and how this tumor stroma-mediated oxidative stress causes a premalignant field defect.

## Results

### Elevated ROS in the microenvironment of numerous tumor types

**Figure 1:**
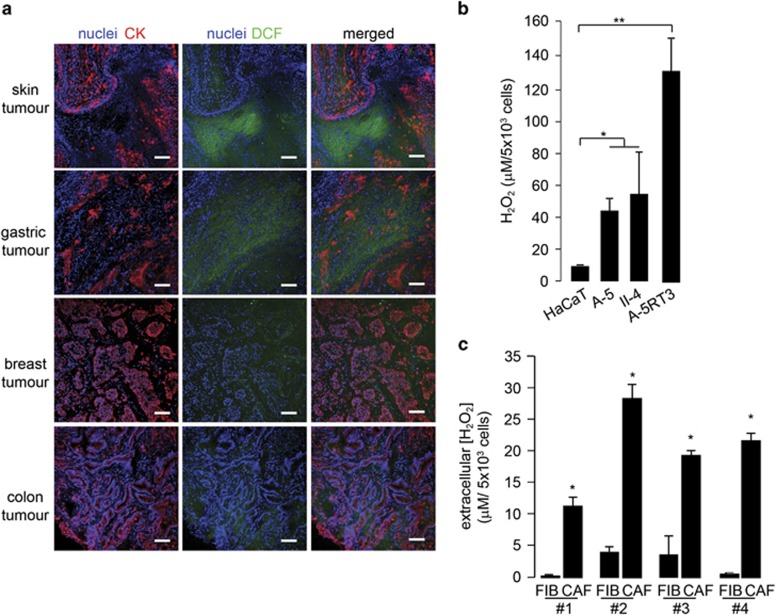
Elevated ROS in the microenvironment of numerous tumor types. (**a**) Dichlorofluorescein diacetate (DCF) staining to detect ROS on sections of patient tumor biopsies from the skin, gastric, breast and colon. Pan-cytokeratin (CK) antibody staining was used to delineate epithelial-stromal boundaries. Sections were counterstained with DAPI. Scale bar: 100 *μ*m. (**b** and **c**) Extracellular concentrations of H_2_O_2_in the conditioned medium from **b**, various human SCC lines, and (**c**) paired FIBs and CAFs from human SCC explants, as measured by Amplex Red H_2_O_2_ assay. *P<0.05, **P<0.01

We first examined the oxidative status of tumor sections from patients with late stage (stage 3 to metastatic) skin squamous cell carcinoma (SCC), gastric, breast and colon cancers using the ROS-sensitive fluorescent dye CM-H_2_DCFDA. Pan-cytokeratin staining distinguished between tumor epithelia and stromal elements. All tumor sections stained positive for ROS (Figure 1a). SCC sections exhibited the highest intensity of ROS staining. Interestingly, ROS were not restricted to the tumor epithelia but were frequently observed in the stromal compartments of tumors. To identify the cell types responsible for elevated microenvironmental ROS, we measured extracellular H_2_O_2_ in conditioned medium from various SCC lines of different aggressiveness (Figure 1b). HaCaT is a non-tumorigenic human keratinocyte cell line, whereas benign A-5, malignant II-4 and highly aggressive A-5RT3 are sublines of different tumorigenic potential derived from HaCaT cells.^16^ HaCaT cells secreted the lowest amount of H_2_O_2_, whereas conditioned medium from A-5RT3 cells had the highest level of H_2_O_2_ (Figure 1b). As CAFs dominate the tumor stroma and diffusible H_2_O_2_ is well suited for a role in tumor stroma crosstalk, we measured the amount of H_2_O_2_ in conditioned medium from CAFs explanted from four patient skin SCC biopsies alongside their paired normal fibroblasts (FIBs) (Figure 1c). We consistently detected higher extracellular H_2_O_2_ in the conditioned medium from SCC CAFs compared with FIBs.

### Microenvironment oxidative stress causes a premalignant field defect

**Figure 2:**
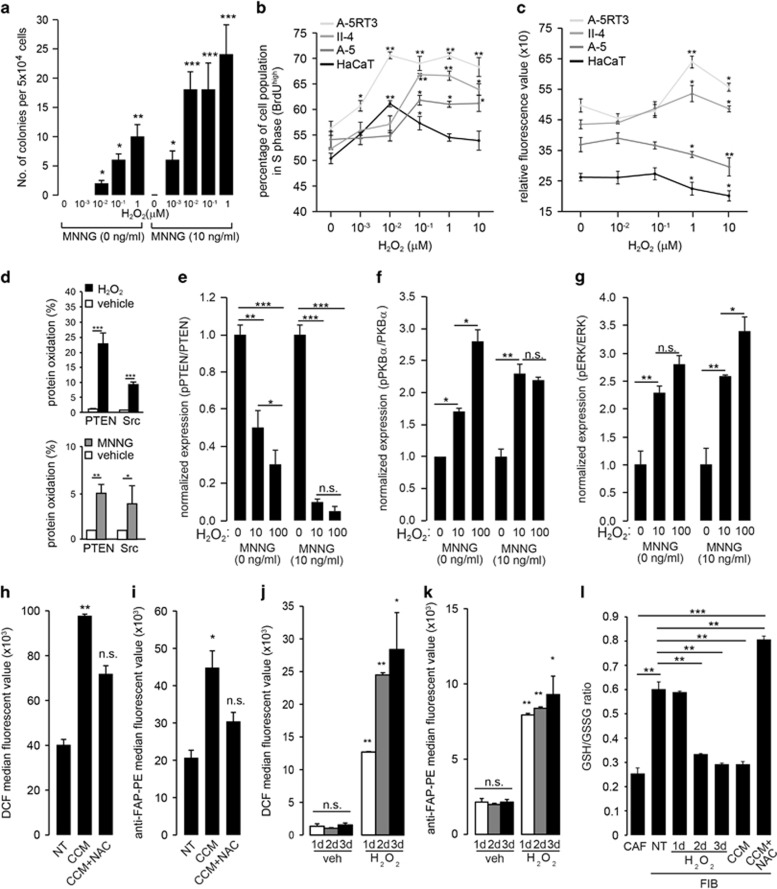
H_2_O_2_ has a transforming effect on epithelia and fibroblasts *in vitro*. (**a**) Quantification of epithelial colonies in soft agar at 1 month after the indicated treatments with MNNG and H_2_O_2_. (**b**) Percentages of proliferating (S-phase) cells after the indicated treatments with H_2_O_2_ as determined by BrdU labeling and FACS analysis. (**c**) Relative fluorescence (SYTO60 dye) readings of invaded cells after the indicated treatments in the transwell invasion assay. (**d**) PEG-switch assay followed by immunoblot analysis of oxidatively modified PTEN and Src in keratinocyte lysates subjected to the indicated treatments. Bars represent the percentage of protein targets that have undergone oxidative modification. (**e**-**g**) Immunoblot analysis of key signaling mediators of the PI3K and ERK pathways in keratinocytes subjected to the indicated treatments. Bars represent normalized densitometry measurements of phosphorylated protein targets normalized against the cognate unphosphorylated forms. (**h**–**k**) Median fluorescence intensities of human fibroblasts subjected to the indicated treatments, followed by staining with DCF and PE-conjugated anti-FAP antibody and FACS analysis. (**l**) Measurements of GSH/GSSG ratio in CAF and FIBs subjected to the indicated treatments. A low GSH/GSSG ratio is an indicator of oxidative stress in cells. The data are presented as the mean±S.D. of three independent experiments. Statistical tests were performed against cognate untreated cells for each cell type. NS, not significant, *P<0.05, **P<0.01, ***P<0.001

Next, we determined whether microenvironmental oxidative stress promotes premalignant development, that is, field cancerization. As the tumor promotion protocol involves a mutagen, *N*-methyl-*N*'-nitro-*N*-nitrosoguanidine (MNNG), we first determined the cytotoxic dose of MNNG in combination with various concentrations of H_2_O_2_. There were no significant differences in the percentages of apoptotic (Annexin V^high^/PI^low^ and Annexin V^high^/PI^high^) and necrotic (AnnexinV^low/^PI^high^) keratinocytes treated with 1–100 nM H_2_O_2_ alone or in combination with 10 ng/ml MNNG (Supplementary Tables S2 and S3). Thus, this range of H_2_O_2_ and MNNG was used to examine the effects of H_2_O_2_ on colony formation in soft agar. A marked increase in colony number was observed with cells pretreated with MNNG and exposed to H_2_O_2_ for 4 weeks (Figure 2a). Keratinocytes treated with a higher concentration of H_2_O_2_ alone formed colonies in soft agar, suggesting that chronic exposure to elevated extracellular H_2_O_2_ facilitates the transformation of epithelial cells. It is conceivable that normal and neoplastic epithelial cells respond differently to H_2_O_2_. Indeed, we observed differential growth response and invasiveness to exogenous H_2_O_2_. The proliferation of HaCaT and A-5RT3 cells peaked at 10 nM H_2_O_2_, whereas the proliferation of A-5 and II-4 cells peaked at 100 nM H_2_O_2_. The proliferation of HaCaT cells began to decrease as the concentration of H_2_O_2_ increased to 10 *μ*M. In contrast, the growth of the three tumorigenic sublines remained significantly elevated compared with untreated cognate controls (Figure 2b). Exogenous H_2_O_2_ also increased the invasiveness of the malignant II-4 and A-5RT3 cells, whereas that of normal HaCaT and benign A-5 cells was reduced as determined by a transwell invasion assay (Figure 2c). The differential growth response and invasiveness was not due to H_2_O_2_-induced apoptosis, as apoptosis of the cell lines was only observed at H_2_O_2_ concentrations >100-fold higher than those used for the proliferation and invasion assays as determined by Annexin V/PI FACS analysis. (Supplementary Figure S1A). These observations indicate that sub-cytotoxic levels of H_2_O_2_ differentially modulate the behavior of normal and neoplastic epithelial cells.

Next, we performed a polyethylene glycol (PEG)-switch assay on H_2_O_2_- and MNNG-treated keratinocytes to identify oxidatively modified proteins. As an oxidant, H_2_O_2_ oxidizes cysteine residues in proteins and modifies their activities. The PEG-switch assay results in the conjugation of PEG to the oxidized cysteine residue, thereby increasing the molecular weight of the oxidized protein and slowing its migration within an SDS-PAGE gel. Proteins bordering the cell membrane are particularly susceptible to modification by extracellular H_2_O_2_. Phosphatase and tensin homolog (PTEN) and Src are well-established mediators of tumorigenesis that are membrane associated. The oxidation of PTEN forms a disulfide bridge that inactivates its tumor-suppressive catalytic activity;^17, 18^ Src oxidation increases its autophosphorylation and concomitant activation.^19, 20^ Indeed, an elevated level of oxidized PTEN and Src in keratinocytes treated with H_2_O_2_ and MNNG was detected by PEG-switch assay, further confirmed by Oxyblot (Figure 2d, Supplementary Figures S1B and C). MNNG alone only contributed to ≤4% of oxidized PTEN and Src, whereas H_2_O_2_ contributed to ~20% oxidized PTEN and ~10% oxidized Src (Figure 2d). Notably, oxidized JNK and EGFR were also detected by PEG-switch, reflecting the promiscuous nature of H_2_O_2_ interactions (Supplementary Figure S1D). PTEN and Src participate in the activation of the PI3K/PKB*α* and MAPK/ERK signaling pathways, which have pivotal roles in tumor development and progression. Immunoblot analysis confirmed increased activation of downstream mediators of the PI3K/PKB*α* and MAPK/ERK pathways (Figures 2e and g, Supplementary Figure S1E). A focused qPCR array comparing H_2_O_2_-treated epithelia and those cocultured with CAFs revealed similar altered expression profiles of many pro-oncogenic and pro-tumor genes. Similarly, the expression of tumor suppressors and growth inhibitory mediators was reduced (Supplementary Figure S1F).

In addition to epithelial cells, H_2_O_2_ may promote field cancerization by affecting adjacent FIBs. We first investigated whether CAF-conditioned medium (CCM) alters the behavior of FIBs. FIBs cultured in CCM had increased ROS levels and elevated expression of fibroblast-activated protein (FAP), a biomarker of CAFs (Figures 2h and i, Supplementary Figures S2A and B). This effect can be attributed, partially, to extracellular ROS because H_2_O_2_ was detected in CCM (Supplementary Figure S2C), and the increase in CAF marker expression was attenuated when CCM was pretreated with the antioxidant NAC. Similar experiments were also performed using H_2_O_2_ alone. Normal FIBs exposed to exogenous H_2_O_2_ also displayed elevated ROS and increased FAP levels after 1 day, and *α*SMA expression was increased after 3 days (Figures 2j and k, Supplementary Figure S2D-F). Consistently, analysis of the GSH:GSSG ratio revealed that CAFs and H_2_O_2_- or CCM-treated FIBs experienced oxidative stress (reflected by a low GSH:GSSG ratio) compared with non-treated FIBs. NAC increased the GSH:GSSG ratio of CCM-treated FIBs (Figure 2l). These collective observations suggest that extratumoral H_2_O_2_ is a transformative agent in stromal field cancerization, stimulating genetic and phenotypic changes in the epithelial cells and normal stromal fibroblasts to generate a field effect.

### Oxidative transformation of FIBs to CAF-like cells was attenuated by catalase *in vivo*

**Figure 3:**
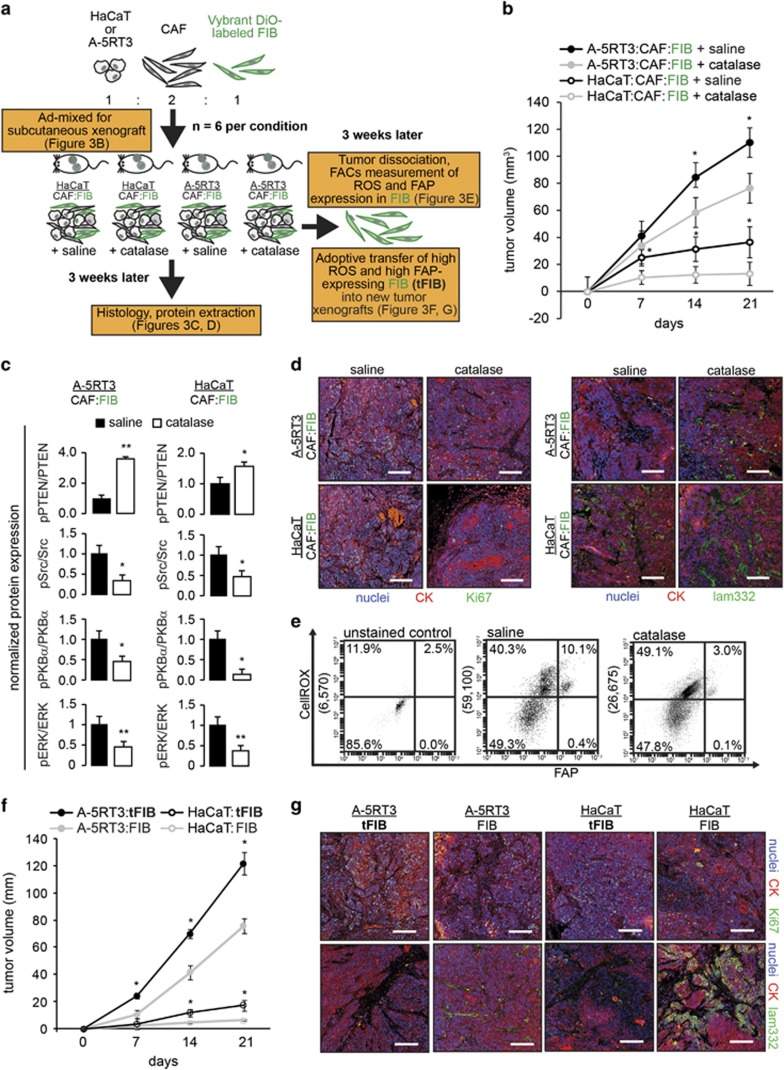
H_2_O_2_ promotes field cancerization *in vivo*. (**a**) Schematic of the experimental approach using VybrantDiO-labeled fibroblasts (FIBs) and CAFs for subcutaneous tumor xenografts in NSG mice. (**b**) Volume measurements of tumor xenografts injected with or without catalase. Statistical tests were performed against cognate saline-treated xenografts. (**c**) Immunoblot analysis of phospho-PTEN, phospho-Src and downstream signaling mediators from tumor lysates. Bars represent normalized densitometry measurements of phosphorylated protein targets normalized against the cognate unphosphorylated forms. (**d** and **g**) Immunofluorescence staining for the proliferation marker Ki67 and the basal lamina protein laminin 332. Pan-cytokeratin (CK) antibody staining was used to delineate epithelial-stromal boundaries. Sections were counterstained with DAPI. Scale bar=100 *μ*m. (**e**) Representative scatterplot for tumor cells stained with CellROX and PE-conjugated FAP antibody. Only VybrantDiO-labeled FIBs were gated for analysis of ROS and FAP expression. The percentage of cells in each quadrant is indicated. The numbers in brackets on the *Y* axis indicate CellROX signal intensity. (**f**) Volume of tumor xenografts with adoptively transferred ROS^high^/FAP^high^tFIBs and untransformed ROS^low^/FAP^low^FIBs. Mice, *n*=6 per experimental condition. The data are presented as the mean±S.D. *P<0.05, **P<0.01

To determine whether extratumoral H_2_O_2_ generates a field effect *in vivo*, we generated an admixed tumor xenograft containing three different cell types, namely, epithelial cells, CAFs and VybrantDiO-labeled FIBs, in the presence or absence of 500 U/ml extracellular catalase (Figure 3a). Cancerous A-5RT3 cells with CAFs:FIBs formed large tumors, whereas non-tumorigenic HaCaT cells with CAFs:FIBs formed small palpable neoplasms (Figure 3b). Notably, tumor growth was attenuated by catalase in the microenvironment (Figure 3b). Consistent with the *in vitro* observations, the tumor xenografts showed reduced activation of key mediators of the PI3K/PKB*α* and ERK pathways when catalase was present (Figure 3c, Supplementary Figure S3A). The number of Ki67-positive proliferating epithelial cells was also reduced in the presence of catalase compared with the saline control (Figure 3d). Depletion of microenvironmental H_2_O_2_ by catalase partially restored basement membrane integrity between the epithelial layer and the adjacent stroma, as shown by a clear laminin 332 staining pattern, suggesting a reduced risk of malignancy (Figure 3d). To determine whether extratumoral H_2_O_2_ transforms normal FIBs into a CAF-like phenotype *in vivo*, we examined intracellular ROS levels and FAP expression in VybrantDiO-labeled FIBs isolated from tumor xenografts by FACS analysis (Supplementary Figure S3B). We observed that 10% of the cells were ROS^high^/FAP^high^FIBs, herein referred to as transformed FIBs (tFIBs), compared with only 3% tFIBs in the presence of catalase (Figure 3e). Next, we asked whether the tFIBs had tumor-promoting capabilities compared with untransformed FIBs (ROS^low^/FAP^low^). To this end, we performed an adoptive transfer of tFIBs and untransformed FIBs into new tumor xenografts (Figures 3f and g). We observed rapid xenograft growth when cancerous A-5RT3 cells were co-injected with tFIBs compared with FIBs. Non-tumorigenic HaCaT cells also grew at an accelerated rate when co-injected with tFIBs compared with FIBs (Figure 3f). Immunofluorescence staining further confirmed a more proliferative and aggressive epithelial cell phenotype in the presence of tFIBs (Figure 3g). Moreover, adoptively transferred tumors comprising A-5RT3 cells and tFIBs exhibited slower growth and less invasion in the presence of extracellular catalase compared with controls (Supplementary Figure S3C). Taken together, our *in vitro* and *in vivo* observations suggest that ROS, specifically H_2_O_2_, induce an oxidative CAF-like state in FIBs for stromal-mediated field cancerization.

### Impaired TGF*β* signaling reduces GPX1 expression in fibroblasts to cause a defect in ROS detoxification

**Figure 4:**
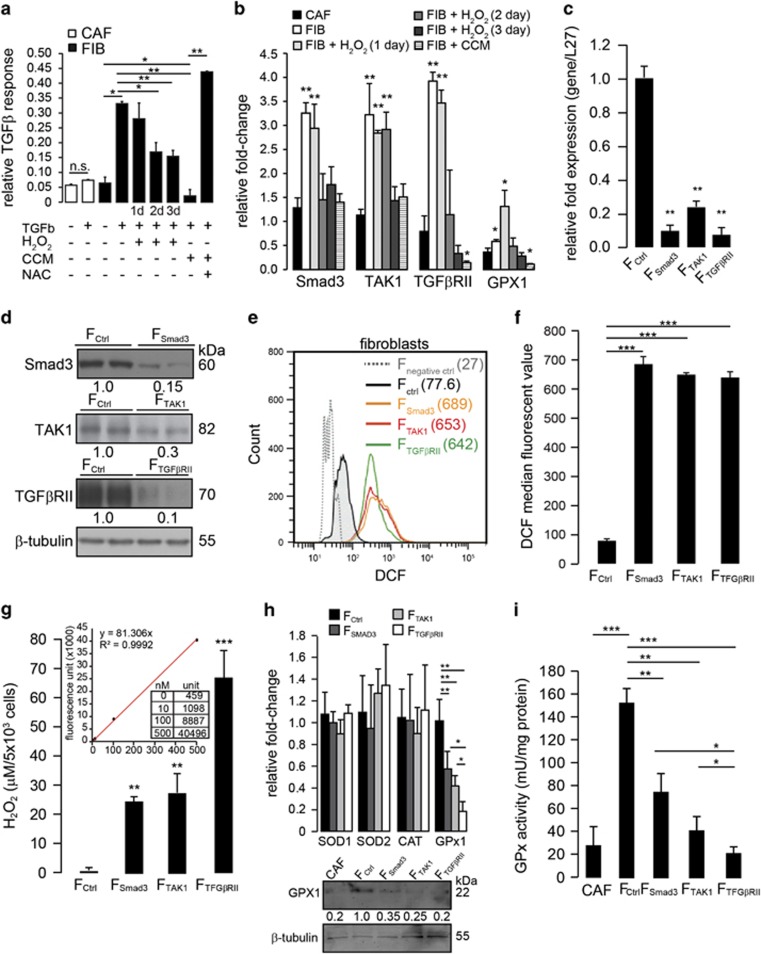
Impaired TGF*β* signaling reduces GPX1 expression in FIBs to cause a defect in ROS detoxification. (**a**) Relative TGF*β*-responsive reporter (luciferase) readings of FIBs and CAFs subjected to the indicated treatments followed by assay for TGF*β* response. (**b** and **c**) Relative Smad3, TAK1, TGF*β*RII and GPX1 mRNA levels in FIBs and CAFs after the indicated treatments. The ribosomal RNA gene Rpl27 served as the housekeeping gene. (**d**) Immunoblot for Smad3, TAK1 and TGF*β*RII protein expression in control (scrambled) FIBs and siRNA knockdown FIBs. *β*-Tubulin from the same samples was used as a loading and transfer control. (**e** and **f**) Levels of ROS in FIBs with siRNA knockdown of Smad3, TAK1 and TGF*β*RII as determined by DCF staining and FACS. (**e**) The values in brackets indicate the mean fluorescence signal intensities. (**f**) Median DCF fluorescence intensity. (**g**) Extracellular H_2_O_2_ in conditioned medium from various knockdown FIBs as measured by Amplex Red assay. The figure insert shows the calibration curve for determining H_2_O_2_ concentration. (**h**) Relative SOD1, SOD2, CAT and GPx1 mRNA levels in knockdown FIBs. Rpl27 served as the housekeeping gene. Immunoblot of the GPx1 protein from the indicated cell lysates. *β*-Tubulin from the same samples was used as a loading and transfer control. (**i**) GPx1 activity measurements for the indicated cells normalized against total protein in the cell lysates. The data are presented as the mean±S.D. of three independent experiments. *P<0.05, **P<0.01, ***P<0.001

Next, we investigated how extratumoral H_2_O_2_ activated FIBs to participate in field cancerization. TGF*β* is a potent mediator of FIB activation, which signals through the canonical Smad and non-canonical TGFβ-activated kinase-1 (TAK1) pathways. We showed that exogenous H_2_O_2_ had similar activating effects on FIBs, such as elevated ROS levels, increased FAP and *α*SMA expression and reduced GSH:GSSG ratio (Figures 2j and l, Supplementary Figure S2F). Next, we probed the effect of H_2_O_2_ on fibroblast TGF*β* signaling. A luciferase assay for TGF*β* response revealed that CAFs have impaired TGF*β* signaling, and FIBs became desensitized to TGF*β* after 2 and 3 days of H_2_O_2_ treatment (Figure 4a). Correspondingly, Smad3 and TGF*β*RII mRNA expression in FIBs decreased after 2 and 3 days of H_2_O_2_ treatment, and TAK1 mRNA expression decreased after 3 days of H_2_O_2_ treatment (Figure 4b). FIBs treated with CCM showed similar impairment of TGF*β* signaling, which was rescued by treatment with NAC (Figures 4a and b).

Oxidative stress also triggers NF*κ*B activity in fibroblasts, leading to a CAF-like phenotype.^21, 22^ Indeed, FIBs treated with exogenous H_2_O_2_ displayed a significant increase in p65-NF*κ*B phosphorylation after 2 and 3 days of H_2_O_2_ treatment (Supplementary Figure S3D). siRNA silencing of p65-NF*κ*B expression in FIBs partially abrogated the H_2_O_2_-mediated suppression of Smad3, TAK1 and TGF*β* mRNA and protein expression (Supplementary Figures S3E-G). These findings suggest that FIBs exposed to H_2_O_2_ initially remain responsive to TGF*β* and this facilitates their activation and acquisition of FAP expression at an early time point, after just 1 day of H_2_O_2_ treatment. Simultaneously, H_2_O_2_ triggers NF*κ*B activation that negatively regulates TGF*β* signaling while potentiating FIB activation. NF*κ*B activation is elevated after 2 days of H_2_O_2_ exposure, thus NF*κ*B maintains FAP expression (and therefore, the CAF-like state) in H_2_O_2_-treated FIBs at later time points when TGF*β* signaling becomes impaired.

To determine whether FIBs with impaired TGF*β* signaling promote the growth of adjacent epithelia, we performed siRNA knockdown of Smad3 (F_Smad3_), TAK1 (F_TAK1_) and TGF*β*RII (F_TGF*β*RII_) in FIBs and measured mitogenic factor secretion and ROS levels. The efficiency of siRNA knockdown was validated by qPCR and immunoblotting (Figures 4c and d). Compared with F_ctrl_, no differences in proliferation or apoptosis of knockdown fibroblasts were observed by FACS analysis (Supplementary Figure S4A). We examined the secreted growth factor protein profile in CCM from these knockdown fibroblasts by ELISA. Out of a total of 76 distinct proteins screened, the expression levels of 7 secreted factors were elevated (Supplementary Figure S4B). A comparative analysis revealed that these factors were differentially modulated by Smad3 and TAK1 in FIBs (Supplementary Figure S4B). To further strengthen our observation, focused real-time PCR arrays comparing epithelia cocultured with CAFs and the various knockdown FIBs were performed. Notably, the expression profiles of many pro-oncogenic or tumor-associated genes in the epithelia cocultured with CAFs were also observed in the epithelia cocultured with F_Smad3_, F_TAK1_, F_TGF*β*RII_ or treated with H_2_O_2_ (Supplementary Figure S1F). Importantly, immunoblot analysis of the epithelial layers of organotypic cocultures of keratinocytes with various knockdown fibroblasts showed the activation of the PI3K/PKB and ERK pathways associated with elevated epithelial proliferation, as indicated by PCNA (Supplementary Figure S4C). Next, we measured ROS levels in knockdown fibroblasts. Compared with F_ctrl_, ROS levels were elevated in F_Smad3_ (8.9-fold), F_TAK1_ (8.4-fold) and F_TGF*β*RII_ (8.2-fold) (Figures 4e and f). We also detected a significant increase in extracellular H_2_O_2_ from the knockdown fibroblasts compared with F_ctrl_. The concentration of extracellular H_2_O_2_ was derived using a calibration curve created using known H_2_O_2_ concentrations (Figure 4g). F_TGF*β*RII_ exhibited the highest amount of extracellular H_2_O_2_ (Figure 4g).

To investigate how impaired TGF*β* signaling increases H_2_O_2_ levels in FIBs, we examined the expression of key enzymes affecting H_2_O_2_ detoxification in FIBs, namely, catalase, superoxide dismutase (SOD) and glutathione peroxidase (Gpx), using real-time PCR. No significant changes were observed in the mRNA levels of SOD1, SOD2 or catalase (Figure 4h). Interestingly, the mRNA and protein expression levels of Gpx1 were reduced in F_Smad3_, F_TAK1_ and F_TGF*β*RII_ compared with F_ctrl_. H_2_O_2_ treatment of FIBs also resulted in an initial spike followed by a gradual loss of Gpx1 expression, corresponding to impaired TGF*β* signaling (Figure 4b). Consistent with the qPCR data, Gpx protein expression and activity were reduced in CAFs and fibroblasts with impaired TGFβ signaling, suggesting that the *GPx1* gene is regulated by Smad3- and TAK1-mediated pathways (Figures 4h and i). These observations indicate that epithelial cells are bathed in an oxidative microenvironment supplemented with mitogenic factors secreted by stromal fibroblasts with impaired TGF*β* signaling.

### Regulation of Gpx1 by cJUN and Smad3 in CAFs from SCCs

**Figure 5:**
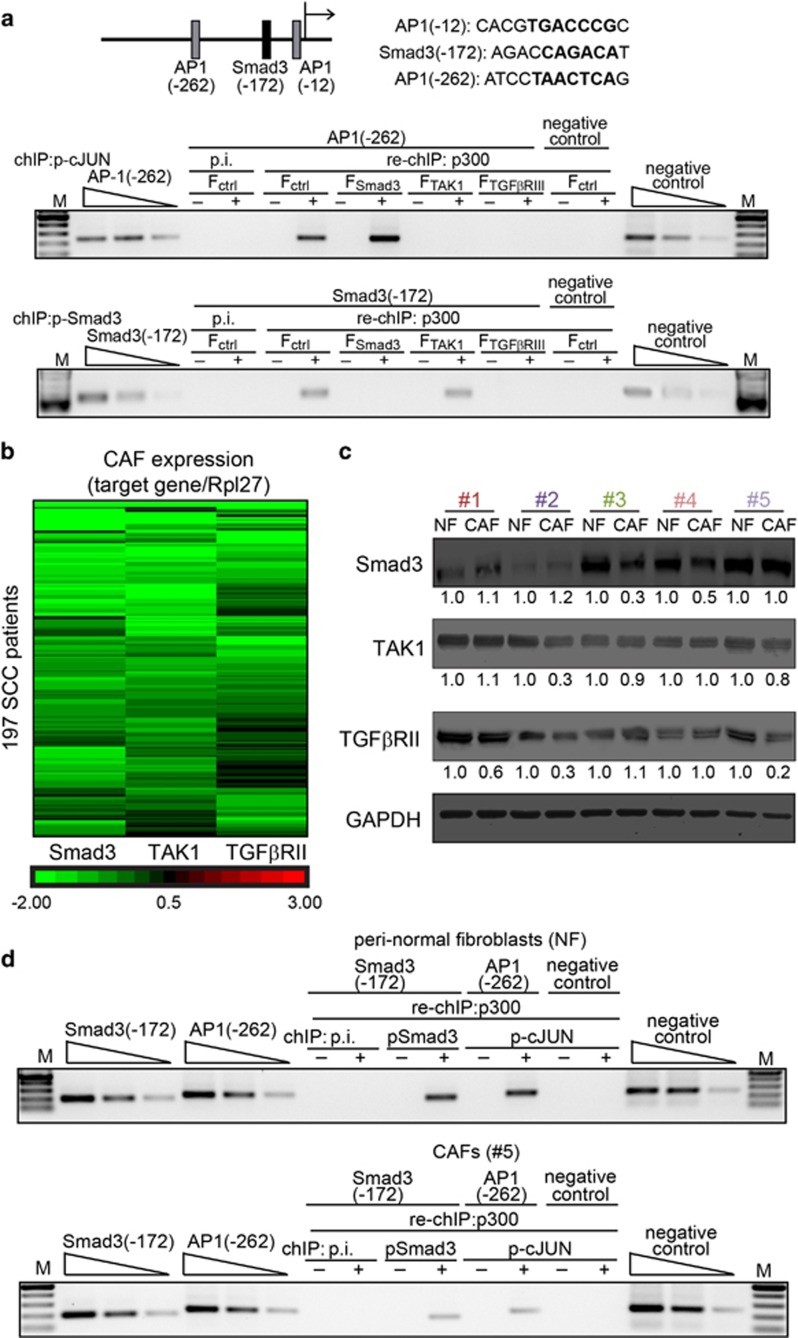
Regulation of Gpx1 by cJUN and Smad3 in CAFs from skin SCCs. (**a**) Schematic of the human *Gpx1* regulatory region showing the positions of the two putative AP1-binding sites and a Smad3-binding site relative to the transcription start site. The sequences of the sites are shown below. Representative ChIP results using anti-phospho-cJUN and anti-phospho-Smad3 antibodies followed by re-ChIP for anti-p300 in F_Smad3_, F_TAK1,_ F_TGF*β*RII_ and F_ctrl_. Preimmune serum (p.i.) was used as the control for ChIP. −/+ Indicate vehicle and TGF*β* treatment, respectively. The data are representative of *n*=3 independent experiments. (**b**) Relative mRNA levels of TGF*β* signaling mediators in a cohort of 197 patients. Fold changes are relative to the cognate NFs. Rpl27 served as the housekeeping gene. The qPCR data shown are from *n*=3 experiments performed in triplicate. (**c**) The relative protein levels of TGF*β* signaling mediators in paired CAFs and NFs from five human SCCs selected at random from the available sample pool for protein analysis. The immunoblot data are from three independent experiments performed in duplicate. GAPDH from the same samples served as a loading and transfer control. The values represent the mean fold differences compared with the cognate NFs. (**d**) Representative ChIP results using anti-phospho-cJUN and anti-phospho-Smad3 antibodies, followed by re-ChIP for anti-p300 using NFs and CAF#5. P.i. was used as the control for ChIP. The data are representative of *n*=3 independent experiments. CAF#5 showed reduced TGF*β*RII expression

*In silico* analysis of the human *GPx1* proximal promoter revealed two putative AP1-binding sites and one SMAD3-binding site (Figure 5a).^23, 24^ TAK1 is also a strong activator of cJun N-terminal kinase, which consequently activates cJUN. To determine whether *GPx1* is a target gene of cJUN and Smad3, chromatin immunoprecipitation (ChIP) experiments for cJUN and Smad3 followed by re-ChIP for p300, a cJUN and Smad3 coactivator, were performed. No binding of phospho-cJUN was detected at AP1(−12), indicating that this binding site is non-functional (Supplementary Figure S5A). There was amplification of the AP1(−262)-binding site in the human *GPx1* promoter after ChIP for phospho-cJUN (Supplementary Figure S5A) and re-ChIP for p300 in F_ctrl_ and F_Smad3_, but not in F_TAK1_ and F_TGF*β*RII_ (Figure 5a). There was also amplification of the Smad3(−172)-binding site after ChIP for phospho-Smad3 (Supplementary Figure S5A) and re-ChIP for p300 in F_ctrl_ and F_TAK1_, but not in F_Smad3_ and F_TGF*β*RII_ (Figure 5a). No binding was detected with preimmune serum, and there was no binding to a control sequence ~2-kb upstream of the transcriptional start site. These observations indicate that the expression of the human *GPx1* gene in fibroblasts is regulated by canonical and non-canonical TGFβ-mediated signaling.

To underscore the clinical relevance of TGF*β* signaling in stromal fibroblasts, we examined the mRNA expression of Smad3, TAK1 and TGF*β*RII in laser-microdissected CAFs from human SCC and adjacent peri-normal fibroblasts (NFs) from 197 patients. During microdissection, we collected the stroma (vimentin-positive) immediately associated with tumor epithelial cells rather than stromal tissue distal to the tumor, and excluded lymphocytes and obvious vascular structures. We observed reduced expression of Smad3, TAK1 and TGF*β*RII, alone or in combination, in CAFs compared with NFs (Figure 5b). Immunoblot analysis of these TGF*β* signaling mediators in CAFs and cognate NFs isolated from five resected SCC samples selected at random from the available patient sample pool showed corresponding changes (Figure 5c). ChIP and re-ChIP experiments were also performed using NFs and CAFs (patient #5; reduced TGF*β*RII expression). Consistent with our earlier observations, the Smad3(-172) and AP1(-262)-binding sites of the human *GPx1* promoter were occupied by Smad3:p300 and cJUN:p300 in NFs, whereas their occupancies were significantly reduced in CAFs (Figure 5d, Supplementary Figure S4B). These results collectively indicate that impaired TGF*β* signaling is a clinical feature of CAFs and is responsible for their loss of *GPx1* expression and activity, which consequently leads to microenvironmental oxidative stress and field cancerization.

## Discussion

CAFs are one of the most abundant stromal cells in the tumor microenvironment, facilitating the development, propagation, and invasiveness of tumors.^25^ Our study showed that CAF-derived ROS, specifically H_2_O_2_, induced oxidative stress in normal FIBs and trigger *de novo* CAF transformation *in vitro* and *in vivo*. Adoptive transfer of oxidatively tFIBs into malignant and non-tumorigenic epithelia xenografts resulted in faster-growing and more aggressive tumors, indicating exacerbated oncogenic transformation. This self-amplifying, field cancerization effect was due to impaired TGF*β* signaling, leading to deficient *GPx1* expression and activity in CAFs. We showed that the human *GPx1* gene was regulated by TGF*β*1-mediated Smad3 and TAK1/cJUN pathways in fibroblasts. Reduced expression of Smad3, TAK1 or TGF*β*RII was also detected in CAFs from a cohort of 197 SCC patients.

Fibroblast-specific deletion of TGF*β*RII in mice led to spontaneous tumors in the prostate and forestomach, which was partially attributed to the activation of paracrine HGF signaling.^26, 27^ However, TGF*β* is required for fibroblast activation and transition to a CAF-like state. To reconcile these paradoxical observations, we noted that TGF*β* signaling operates in a negative feedback loop mediated by the inhibitory Smad, Smad7,^28, 29^ which is upregulated by TGF*β* via the pro-inflammatory transcription factor NF*κ*B.^30, 31^ Oxidative stress is also strongly associated with NF*κ*B activation.^32, 33^ Hence, FIBs exposed to H_2_O_2_ are initially responsive to TGF*β*, which facilitates their activation by tumor epithelia- and CAF-derived TGF*β*. Simultaneously, H_2_O_2_ triggers NF*κ*B activation to suppress TGF*β* signaling and GPx1 expression while potentiating FIB activation. In mature tumors, TGF*β* is abundant and the tumor microenvironment is chronically subjected to low-grade inflammation and redox imbalance. These combined characteristics synergistically create an ecosystem that encourages the conversion of FIBs to CAFs, then renders CAFs refractory to TGF*β* signaling and highly oxidative because of *Gpx1* deficiency. The expression of *Gpx1* is also regulated by p53,^34^ and the ablation or mutation of p53 in stromal fibroblasts increased growth and metastatic spread of PC3 prostate cancer cells because of increased production of SDF-1.^35^ Although the redox status of p53-deficient fibroblasts is unknown, it is conceivable that elevated H_2_O_2_ may contribute to their pro-tumorigenic effect. Together with our own observation that silencing NF*κ*B expression could prevent the H_2_O_2_-mediated impairment of TGF*β* signaling in fibroblasts, these findings suggest that transcriptional control has a critical role in modulating the pro-tumorigenic effects of CAFs.

Recent studies have revealed an important relationship between the extracellular redox state and cancer aggressiveness.^9^ The activation of oncogenes in epithelial cells results in the loss of caveolin-1 and the elevated expression of lactate transporters in stromal fibroblasts. These CAF-like fibroblasts exhibited increased ROS levels and elevated glucose uptake, which complement cancer cell metabolism.^26, 36^ We showed that extratumoral H_2_O_2_, either from epithelial tumor cells or CAFs, reinforced the already dysregulated epithelial–mesenchymal communication to promote field cancerization. Indeed, the prolonged exposure of primary human keratinocytes to low levels of H_2_O_2_ elevated the levels of oxidatively modified PTEN and Src, which triggered oncogenic signaling to promote epithelial transformation and growth; oxidization of the tumor suppressor PTEN and the oncogene Src results in protein inactivation and activation, respectively. H_2_O_2_ can also differentially influence the proliferation and invasiveness of cancer cells at various stages of progression. Lending support to a pro-mitogenic role of H_2_O_2_, H_2_O_2_ antagonized the cytostatic functions of TGF*β*1 by the activation of Akt-ERK1/2-linked signaling.^37^ Although the precise target of H_2_O_2_ was not identified, many studies have shown that Akt-ERK1/2 are downstream mediators of Src and PTEN.^34, 38^ Similarly, the migration of MCF-7 breast cancer cells was enhanced by elevated Nox4 expression in a human mammary fibroblast cell line.^36^ Nox4 is known to predominantly produce H_2_O_2_ rather than O^2−^.^39^

Finally, CAFs exert their proneoplastic activity by secreting mitogenic factors. Many of these factors are potential druggable targets, and it has been proposed that the inhibition of these ligand/receptor-activated signaling networks could limit neoplastic growth. We detected an increase in pro-mitogenic factors such as HGF and FGF7 in the conditioned media harvested from keratinocytes cocultured with fibroblasts with impaired TGF*β*signaling. These elevated mitogenic factors, together with extracellular H_2_O_2,_ promoted cancer cell proliferation, as supported by the activation of pro-oncogenic PKB*α*, ERK and JNK pathways in the epithelia. Our findings that the oxidative action of H_2_O_2_ also enables CAF-mediated field cancerization suggest that anticancer strategies that singularly target the ligand-receptor pathways may not be effective. For example, a neutralizing antibody to HGF exhibited potent antitumor effect in the treatment of glioblastoma multiforme.^40^ However, tumor growth became HGF-independent after repeated treatments.^41^ Conceivably, the promiscuous nature of H_2_O_2_ interactions with cellular signaling components, especially membrane-associated molecules such as EGFR and Met, renders the cells less dependent on ligand-activated pathways to promote transformation and growth. Indeed, initial efforts focused on the inhibition of PDGF with a single agent, imatinib, generated poor outcomes.^42, 43^ In contrast, combination treatment with hydroxyurea and imatinib has provided very encouraging findings.^44, 45^ Interestingly, hydroxyurea is a potent antioxidant that has been shown to increase the expression of *Gpx1* in a p53-dependent manner, among other effects.^46^ Future exploration of new multi-modal approaches that simultaneously target ligand-receptor signaling and redox-mediated pathways will offer new promising anticancer strategies.

## Materials and methods

### Human tumor samples

Human SCC biopsies along with their paired peri-normal tissues were provided by the National Skin Centre, Singapore. CAFs and FIBs were isolated by laser capture microdissection and subjected to protein and RNA extraction for immunoblotting and real-time PCR analyses, respectively.^47, 48^ The study was approved by the National Healthcare Group Domain-Specific Review Boards (NHG-DSRB). All tumor samples were de-identified before the analyses.

### *In vitro* tumor colony promotion protocol

Keratinocytes were seeded at a density of 1 × 10^5^ per 35-mm dish containing complete OTC medium (Denova Sciences, Singapore) at day 0. On day 1, cultures were treated for 1 h with the initiating agent, MNNG, or solvent control (DMSO). The cells were washed, and the medium was replaced with complete medium for 24 h before exposure to different concentrations of H_2_O_2_. After 1 week, cells were harvested from individual dishes and passaged at a density of 1 × 10^5^ per 35-mm dish in H_2_O_2_-containing medium. Concurrently, 2 × 10^4^ harvested cells were embedded in 0.35% noble agar (Sigma Aldrich, St. Louis, MO, USA) and layered onto 0.7% noble agar-coated 35-mm dishes during each passage of the cells. These soft agar cultures were consistently replenished with fresh H_2_O_2_-containing medium every other day. After 1 month, viable transformed colonies were stained with 1 mg/ml thiazolyl blue tetrazolium in PBS and counted.

### Cell proliferation, apoptosis assays and FACS analysis

Bromodeoxyuridine (BrdU) incorporation and total DNA content in proliferating cells were analyzed by BrdU and PI staining using the BrdU Flow kit (BD Biosciences, San Jose, CA, USA). Epithelial cells were treated for 24 h with the indicated concentrations of H_2_O_2_ in serum-free medium. The cells were incubated with BrdU (40 *μ*M) for 30 min. Apoptotic and necrotic cells were quantified using an Annexin V/PI staining kit (BioLegend, San Diego, CA, USA) and subsequent FACS analysis.

### PEG-switch assay and OxyBlot protein oxidation detection

PEG-switch assay was performed as previously described.^49^ Cells treated with 100 nM H_2_O_2_ or 10 ng/ml MNNG for 24 h were lysed using M-PER mammalian protein extraction reagent (Thermo Fisher Scientific, Waltham, MA, USA). Oxidized thiols were reduced using DTT and alkylated using 2 mM PEG-maleimide with 0.5% SDS for 2 h at room temperature. The alkylation reaction was quenched using 100 mM Tris–HCl buffer pH 6.8, 4% SDS, 20% glycerol, and 0.01% bromophenol blue and 100 mM maleimide and the samples were resolved by SDS-PAGE and subjected to immunoblot. Oxidatively modified PTEN was analyzed as previously described with some modifications.^17^ Cells treated with 100 nM H_2_O_2_ for 30 min were washed with oxygen-free ice-cold PBS containing 1 mM *N*-ethylmaleimide (NEM). Cells were lysed in lysis buffer (50 mM Tris, pH 6.8, 150 mM NaCl, 1 mM EDTA, 0.5% Triton X-100 and 30 mM NEM). Lysates were further clarified using aQIAshredder (Qiagen, Valencia, CA, USA). Equal amounts of proteins were mixed with NEM loading dye (50 mM Tris, pH 6.8, 10% glycerol, 2% SDS, 20 mM NEM and 0.02% bromophenol blue) and resolved by non-reducing SDS-PAGE. As a control, proteins were completely reduced by heating in SDS-loading dye containing DTT. Oxidized Src was detected as previously described.^19^ The separated proteins were subjected to immunoblot analysis using antibodies against PTEN and Src.

### H_2_O_2_ and CCM treatment of FIBs *in vitro*

FIBs and CAFs were seeded at a density of 1 × 10^4^ per 35-mm dish in FibroGRO-LS (Millipore, Billerica, MA, USA). The 48- h CCM was collected from CAFs, sterile filtered and used immediately. CCM, with or without 100 *μ*M *N-*acetylcysteine (Sigma Aldrich) pretreatment, was added to wells containing FIBs and refreshed daily over 3 days, after which FIBs were harvested for FACS analysis. Separately, FIBs were treated with 100 nM H_2_O_2_ in serum-free FibroGRO-LS for 30 min twice daily with an 8-h interval between treatments for a maximum of 3 days. FIBs were harvested for FACS analysis 24 h after 1 day, 2 days and 3 days of H_2_O_2_ treatment.

### *In vivo* tumor growth and adoptive transfer of tFIBs

A total of 1 × 10^5^ HaCaT or A-5RT3 cells were admixed with 2 × 10^5^ CAFs and 1 × 10^5^VybrantDiO (Life Technologies, Waltham, MA, USA)-labeled FIBs in growth factor-reduced Matrigel (Corning, Tewksbury, MA, USA), with or without 500 U/ml catalase (Sigma), and implanted subcutaneously into NSG mice (Jackson Laboratory, Bar Harbor, ME, USA) (Figure 3a). Three weeks post-implantation, tumors were excised and dissociated using the MACS tumor dissociation kit and a gentleMACS Octo Dissociator (Miltenyi Biotec, Germany). Then, the tumors were doubly stained with CellROX Deep Red (Thermo, Waltham, MA, USA) and PE-conjugated anti-FAP antibody (Novus Biologicals, Littleton, CO, USA) for FACS. A total of 3 × 10^5^ ROS^high^/FAP^high^tFIBs were isolated and adoptively transferred into new tumor xenografts containing either HaCaT or A-5RT3 cells. Tumor growth was monitored for 3 weeks. All animal studies were approved by the Institutional Animal Care and Use Committee of Nanyang Technological University (ARF-SBS/NIE-A0216AZ).

### ChIP and re-ChIP

ChIP was performed using phospho-cJUN and phospho-Smad3 antibodies (Cell Signaling, Danvers, MA, USA), and re-ChIP was performed using a p300 antibody (Upstate Biotechnology, Lake Placid, NY, USA) as previously described.^50^ The primer sequences are listed in Supplementary Table S1.

### Immunoblot assay

Far-infrared immunoblotting was performed as previously described.^51^

### Measurement of extracellular H_2_O_2_,intracellular ROS and GSH/GSSG ratio

Extracellular H_2_O_2_ and intracellular ROS were measured as previously described.^47, 52^ The specificity of the assay for H_2_O_2_ was verified with catalase, and the degradation of H_2_O_2_ or inhibition of the assay system by the sample was analyzed by determining the recovery of exogenously added H_2_O_2_. GSH/GSSG ratio was measured using the GSH/GSSG ratio detection assay kit green (Abcam, Cambridge, UK) according to the manufacturer's recommendations.

### Mitogenic factor array

The expression profiles of the secreted factors present in the conditioned medium (48 h) were identified using the RayBio Human Growth Factor Array 1 and Human Inflammation Antibody Array 3 (RayBiotech, Inc., Norcross, GA, USA). These membranes were incubated with conditioned media from the different cocultures and then processed.

### Statistical analysis

Statistical differences were determined using a two-tailed Mann–Whitney *U*-test. A *P*-value of <0.05 was considered statistically significant.
