# Supplementary material for: Cancer-associated fibroblasts enact field cancerization by promoting extratumoral oxidative stress
Source: Cell Death Dis. 2017 Jan 19;8(1):e2562–. doi: 10.1038/cddis.2016.492 (PMC5386391; doi:10.1038/cddis.2016.492)
Supplement: Supplementary Information [file cddis2016492x1.docx]

**Cancer-associated fibroblasts enact field cancerization by promoting extratumoral oxidative stress**

Jeremy Soon Kiat CHAN^1^, Ming Jie TAN^1,#^, Ming Keat SNG^1^, Ziqiang TEO^1^, Terri PHUA^1,2^, Chee Chong CHOO^1^, Liang LI^1^, Pengcheng ZHU^1^, Nguan Soon TAN^1,3,4,5^

^1^School of Biological Sciences, Nanyang Technological University, 60 Nanyang Drive, Singapore 637551.

^2^Karolinska Institutet, Department of Microbiology, Tumor and Cell Biology, Nobelsvӓg 16, Stockholm 17177, Sweden.

^3^Lee Kong Chian School of Medicine, Nanyang Technological University, 50 Nanyang Avenue, Singapore 639798.

^4^Institute of Molecular and Cell Biology, A*STAR, 61 Biopolis Drive, Proteos, Singapore 138673.

^5^KK Women’s and Children Hospital, 100 Bukit Timah Road, Singapore 229899.

^#^Present address: DeNova Sciences Pte Ltd, 16 Nanyang Drive, Innovation Centre Blk 1, Singapore 637722.

Running title: Oxidative stress causes a premalignant field defect

Key words: Field cancerization, oxidative stress, cancer associated fibroblasts

The work was supported by grants from the Singapore Ministry of Education (MOE2010-T2-2-009 and AcRF Tier 1 RG134/15) to NST.

Correspondence to JSKC: [CHAN0693@e.ntu.edu.sg](mailto:CHAN0693@e.ntu.edu.sg); PZ: [PCZhu@ntu.edu.sg](mailto:PCZhu@ntu.edu.sg);

NST (senior corresponding author): [nstan@ntu.edu.sg](mailto:nstan@ntu.edu.sg); Tel.: +65-63162941; Fax: +65-67913856

**The authors have no conflicts of interest to declare.**

**SUPPLEMENTARY INFORMATION**

**Supplementary Materials and Methods**

**Supplementary Figure S1.** The effect of H_2_O_2_ on the oncogenic transformation of keratinocytes.

**Supplementary Figure S2.** The effect of H_2_O_2_ on the oncogenic transformation of fibroblasts.

**Supplementary Figure S3.** Catalase inhibition of proliferation and invasiveness in composite tumor xenografts, and H_2_O_2_-mediated downregulation of TGFβ signaling via NFκB activation.

**Supplementary Figure S4.** Effect of TGFβRII, TAK1 and Smad3 knockdown on FIBs proliferation, apoptosis and paracrine signaling with keratinocytes.

**Supplementary Figure S5.** ChIP for Smad3 and p-cJUN occupancy on the Gpx1 promoter of FIBs and CAFs.

**Supplementary Table S1.** List of primer sequences

**Supplementary Table S2.** Percentage of apoptotic cells after exposure to indicated concentrations of MNNG and H_2_O_2_.

**Supplementary Table S3:** Percentage of necrotic cells after exposure to indicated concentration of MNNG and H_2_O_2_.

**Supplementary Materials and Methods**

*Cell culture conditions.*

Human primary keratinocytes and fibroblasts were maintained in Keratinocyte-SFM (Life Technologies) and FibroGRO-LS (Millipore), respectively. Human primary keratinocytes were obtained from ATCC,FIBs and CAFs were purchased fromAsterand Bioscience in 2015. All primary cells were used at low passage numbers (3-5 passages). HaCaT, II-4, A-5 and A-5RT3 (German Cancer Research Center, 2011) were cultured in DMEM supplemented with 10% FBS. Cells were maintained in a 37 ^o^C, 5% CO_2_, humidified incubator. All cell lines were tested to exclude mycoplamsa contamination and authenticated against the ICLAC database.

*siRNA knockdown.*

Fibroblasts were transfected with SMARTpool small interfering RNAs (siRNAs) targeting either TGFβRII, Smad3, TAK1 or NFκB using DharmaFECT1 according to manufacturer’s recommendation (Dharmacon).

*Quantitative real-time PCR (qPCR).*

Total RNA was extracted using Trizol (Life Technologies). qPCR was performed as previously described^1^. Heatmaps were constructed using Orange Canvas software (University of Ljubljana). Primer sequences are in Supplementary Table S1.

*Transwell Invasion Assay*

Transwell inserts (diameter 6.5 mm, pore size 8 μm; Costar) were coated on the upper side with 50 μg/mL of type I collagen (Sigma-Aldrich). Epithelial cells were seeded at a density of 4x10^4^ cells per insert. On the assay day, the upper chamber was washed with PBS and replaced with serum-free DMEM containing various concentrations of H_2_O_2_. DMEM supplemented with 5% FBS was added into well to provide the chemotactic gradient. Culture inserts were incubated at 37 ^o^C, 5% CO_2_ for 14 h in a humidified incubator. Cells in the upper chamber were removed with a cotton swab. Invaded cells remaining on the bottom of the inserts were stained with infrared dye, 1 µM SYTO^®^60 (Life Technologies) in PBS for 5 min at 37 ^o^C. SYTO^®^60 is cell permeant and fluoresces at 680 nm only when bound to cellular DNA. Fluorescence of each insert was read using the Odyssey CLx infrared imaging system (LI-COR Biosciences).

*Organotypic coculture (OTC).*

OTC was performed as previously described^2^.

**Supplementary Figures**

**
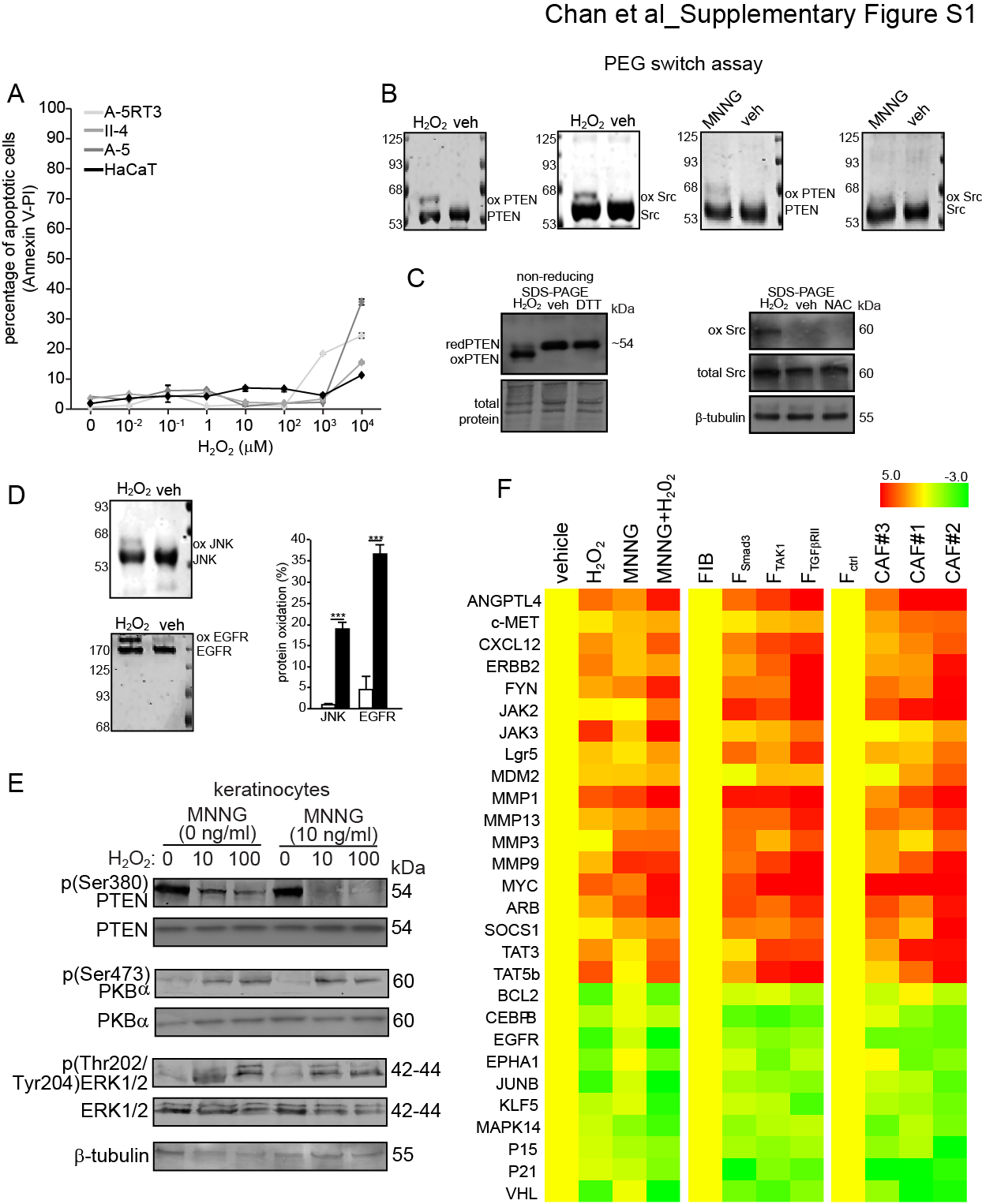
**

**Supplementary Figure S1.** The effect of H_2_O_2_ on the oncogenic transformation of keratinocytes. A, FACS analysis of the epithelial cell lines subjected to the indicated concentrations of H_2_O_2_ treatments for 24 h, then stained using Annexin-V FITC antobodies and propidium iodide to identify apoptotic cells. B,D, PEG-switch assay immunoblots for oxidized PTEN, Src, JNK and and EGFR proteins in keratinocytes treated with 100 nM of H_2_O_2_ or 10 ng/mL of MNNG Oxidized proteins become conjugated with PEG and have a higher molecular weight than their unoxidized couterparts. Bar charts represent the percentage of protein targets that have undergone oxidative modification. C, Oxyblot for oxidized PTEN and Src proteins in keratinocytes treated with 100 nM of H_2_O_2_ or 10 ng/mL of MNNG. Oxidized PTEN protein migrates faster under non-reducing SDS-PAGE conditions compared to its reduced counterpart. E, Immunoblot analysis for key signaling mediators of PI3K and ERK pathways of keratinocytes subjected to the indicated treatments. Phosphorylated protein targets were normalized against the cognate unphosphorylated forms from the samples. F, Focus qPCR array on keratinocytes with the indicated treatments (left panel) or in coculture with the indicated FIBs (middle panel) and CAFs (right panel).


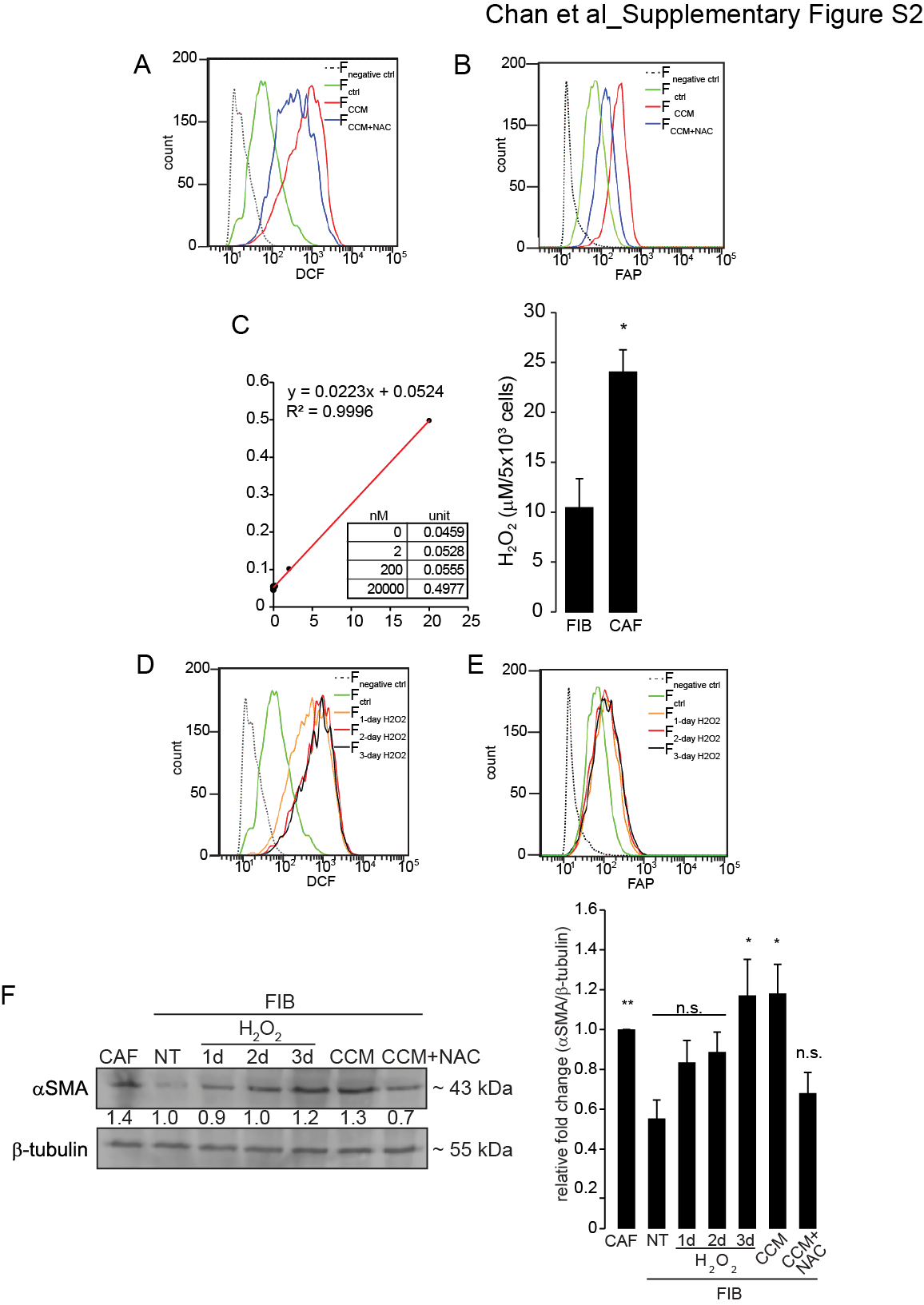


**Supplementary Figure S2.** The effect of H_2_O_2_ on the oncogenic transformation of fibroblasts. A-B, D-E, Representative FACs histograms of human fibroblasts subjected to the indicated treatments followed by staining with DCF and PE-conjugated anti-FAP antibody. C, Calibration curve for determination of H_2_O_2_ concentration (left). Extracellular concentration H_2_O_2_ in conditioned medium of FIBs and CAFs measured by Amplex Red H_2_O_2_ assay (right). Data, means ± S.D. from 3 independent experiments. *p<0.05, **p<0.01. H, Immunoblot of αSMA from lysates of CAFs and FIBs subjected to the indicated treatments. β-tubulin was used as loading and transfer control from the same samples. F, Densitometry measurements of immunoblot for αSMA from lysates of CAFs and FIBs subjected to the indicated treatments. β-tubulin from the same samples was used as a loading and transfer control.


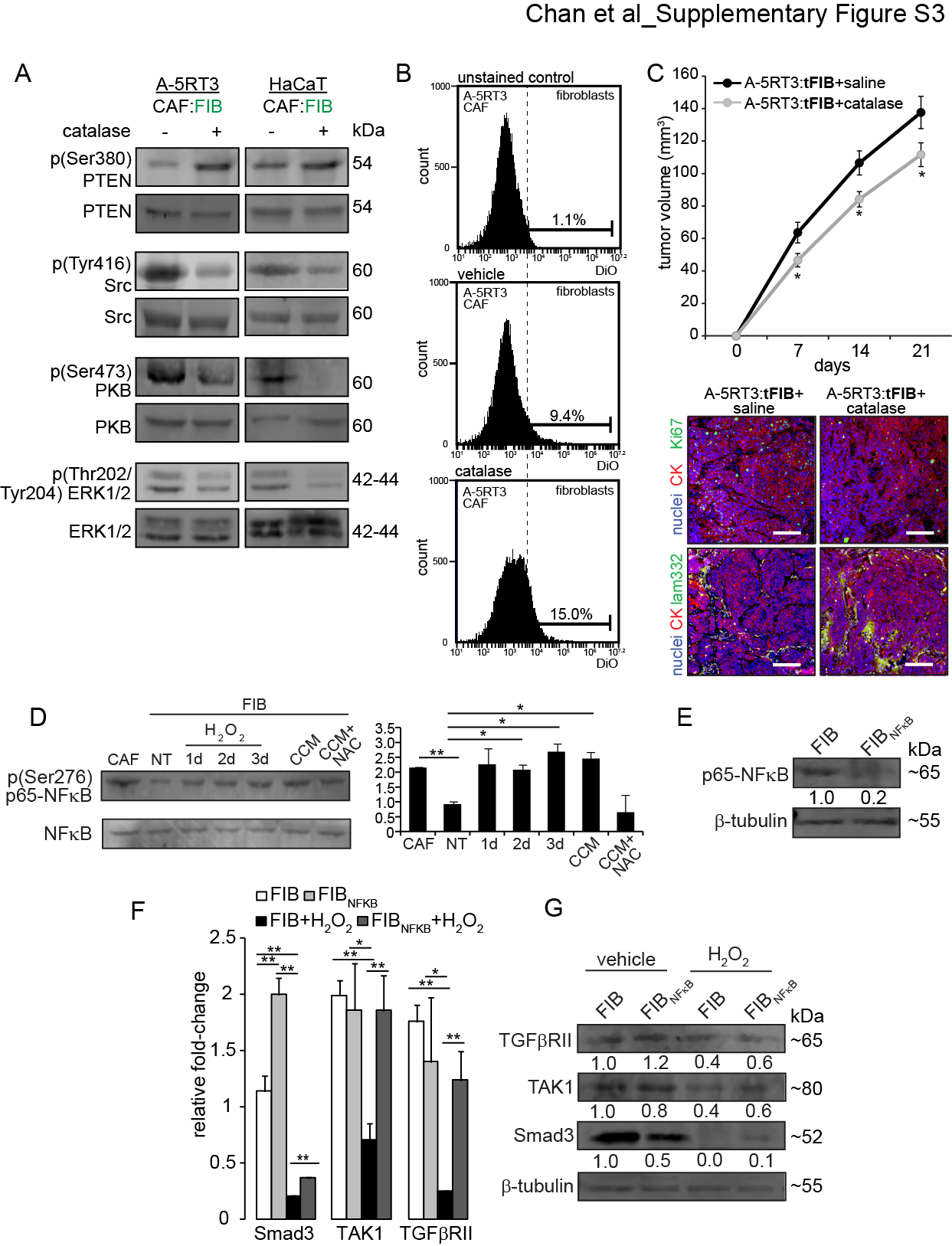


**Supplementary Figure S3.** Catalase inhibition of proliferation and invasiveness in composite tumor xenografts, and H_2_O_2_-mediated downregulation of TGFβ signaling via NFκB activation. A, Immunoblot of phospho-PTEN, phospho-Src and downstream signaling mediators from tumor lysates.β-tubulin from the same samples was used as loading and transfer control.). B, Flow cytometry gating of Vybrant DiO-labelled FIBs recovered from tumor xenografts with or without catalase. Isolated FIBs were analyzed for ROS and FAP expression followed by adoptive transfer of ROS^high^FAP^high^tFIBs and untransformed ROS^low^FAP^low^FIBs into new tumor xenographs. C,Volume measurements of tumor xenografts injected with or without catalase (n = 6 per experimental condition). Statistical tests were performed against cognate saline-treated xenografts.Immunofluorescence staining for the proliferation marker Ki67 and the basal lamina protein laminin 332. Pan-cytokeratin (CK) antibody staining was used to delineate epithelial-stromal boundaries. Sections were counterstained with DAPI. Scale bar = 100 µm. D, Immunoblot for p65-NFκB and phospho-p65-NFκB expression in FIBs subjected to the indicated treatments. Bars represent normalized densitometry measurements of phosphorylated protein targets normalized against the cognate unphosphorylated forms. E, Immunoblot for p65-NFκB in FIBs andp65-NFκB-knockdown FIBs (FIB_NFκB_). β-tubulin from the same samples was used as loading and transfer control.F-G, Relative mRNA and protein levels of Smad3, TAK1 and TGFβRII in FIBs and FIB_NFκB_subjected to the indicated treatments. Ribosomal RNA gene Rpl27 was used a normalizing housekeeping gene. β-tubulin from the same samples was used as loading and transfer control. Data from 3 independent experiments.

**
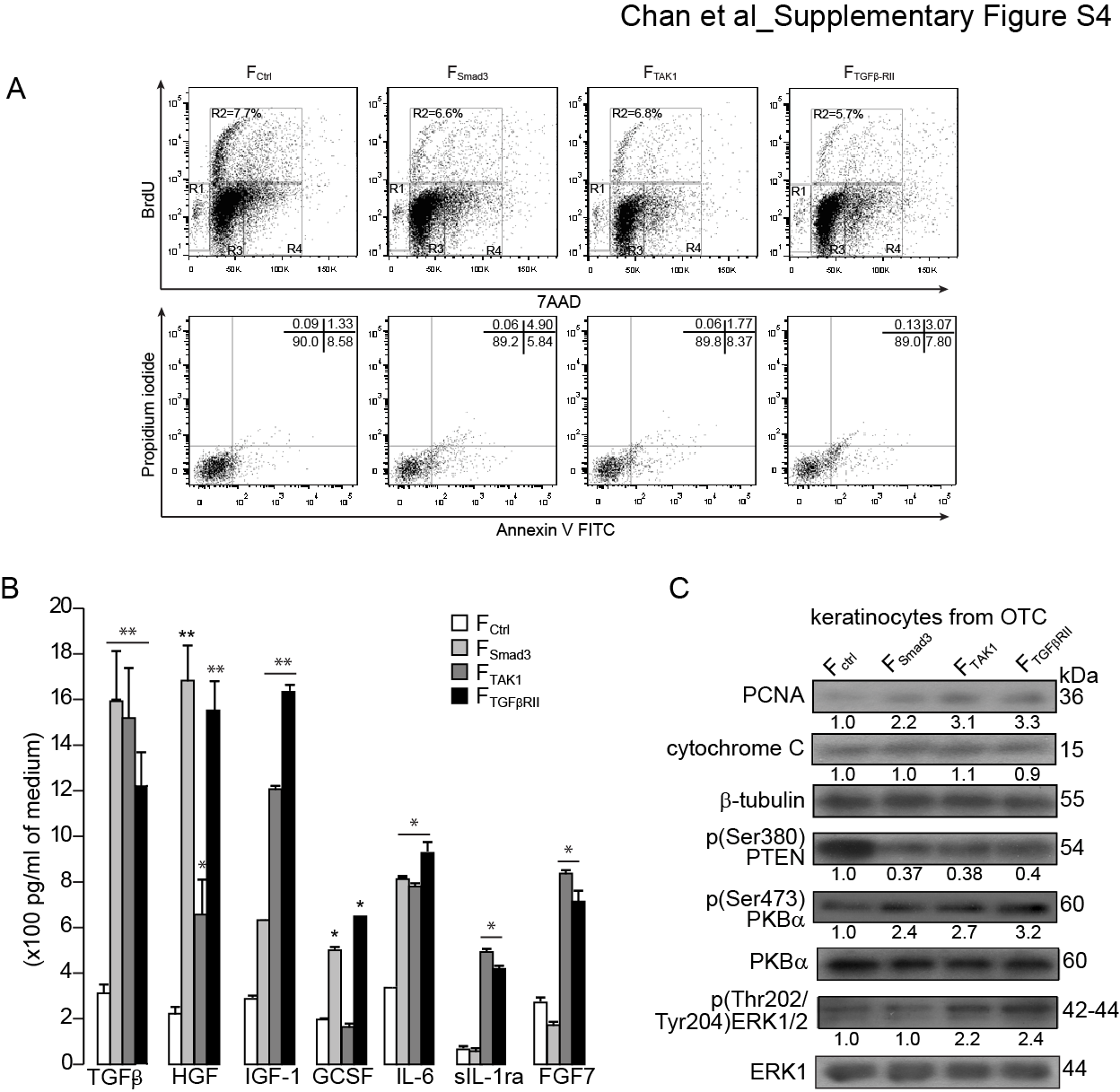
**

**Supplementary Figure S4.** Effect of TGFβRII, TAK1 and Smad3 knockdown on FIBs proliferation, apoptosis and paracrine signaling with keratinocytes. A, FACs analysis of BrdU (top panel) and Annexin-PI (bottom panel) for proliferation and apoptosis status in knockdown FIBs. B, Mitogenic factor array in the 48 h conditioned medium from FIBs or knockdown FIBs. The data are presented as the mean ± S.D. of 3 independent experiments. Statistical tests were performed against F_ctrl_. **p*<0.05, **p<0.01. C, Immunoblot for the indicated proteins in keratinocytes from OTCs cocultured with FIBs or indicated knockdown FIBs. β-tubulin from the same samples was used as loading and transfer control.

**
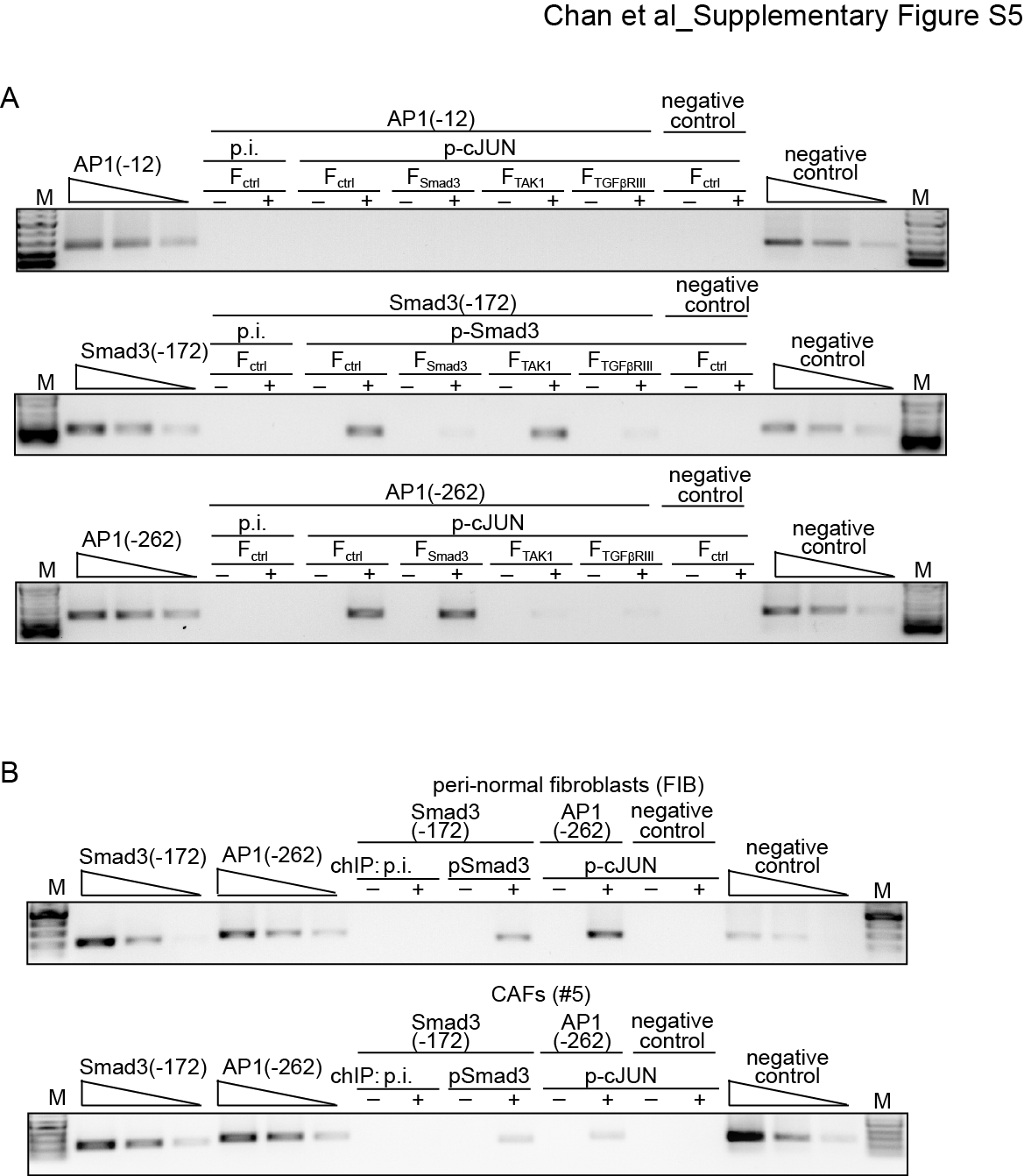
**

**Supplementary Figure S5.** ChIP for Smad3 and p-cJUN occupancy on the*Gpx1* promoter of FIBs and CAFs.Representative ChIP results using anti-phospho-cJUN and anti-phospho-Smad3 antibodies performed in F_Smad3_, F_TAK1,_ F_TGFβRII_ and F_ctrl_. Preimmune serum (p.i.) served as control for ChIP. -/+ indicate vehicle and TGFβ treatment respectively. Data are representative of n=3 independent experiments.

**Supplementary Table S1:** List of primer sequences

| **ChIP primers** | **Forward (5’ to 3’)** | **Reverse (5’ to 3’)** |
| --- | --- | --- |
| AP1(-12) | TTCCGGCTTAGGAGGAGCAC | CCGCCGCTAGCCGAGCAGC |
| Smad3(-171) | GGAACCTCTGAGAAAAAA | TGAGGGTCCCGCCTCATCCG |
| AP1(-262) | ACCGGAAGCCTAGATCCCTCTG | TCCAGGTTACCCTTCCAAGCTG |
| Negative control (-2016) | AGGCTGGGTACAGTGGCTCATG | GCTCGTCGGTCGCCCAGGC |
| **qPCR primers** |  |  |
| ANGPTL4 | CTCCCGTTAGCCCCTGAGCG | AGGTGCTGCTTCTCCAGGTG |
| BCL-2 | GAACTGGGGGAGGATTGTGG | CCGGTTCAGGTACTCAGTCA |
| C/EBPβ | CTTCAGCCCGTACCTGGAG | GGAGAGGAAGTCGTGGTGC |
| cMET | TGAAGTAATGCTAAAATGCTGG | CCTATGGCAAGGAGCAAAGA |
| CXCL12 | CAGCCTGAGCTACAGATGCC | CTGAAGGGCACAGTTTGGAG |
| EGFR | AAGGAAATCCTCGATGAAGCCT | TGTCTTTGTGTTCCCGGACATA |
| EPHA1 | CAAGGACGCAGAGACACTGAC | CACCGTGGTTACCTTCTGGA |
| ERBb2 | CTCTTAGACCATGTCCGGGAA | AGCCCGAAGTCTGTAATTTTGAC |
| FYN | TTTCAATTCCGTAGCCAGCTGC | ACAGAGGAGGTTCGGATTTGGG |
| JAK2 | GTATCCACCCAACCATGTCTTC | GTTGCTGCCACTGCAATACC |
| JAK3 | AGGACCGGCCATCATTCAG | AGCAGTGAAGGCATGAGTCTC |
| JUN-B | TACCACGACGACTCATACACA | CGCTTTGAGACTCCGGTAGG |
| KLF-5 | CTGAGAACTGGCCTCTACAAATC | TGGTGTGAGCTGAATATACTGGT |
| Lgr5 | ACGCTCTGACATACATTCCCA | GATTGAAGGCTTCGCAAATTCTG |
| MAPK14 | CAGCAGATTATGCGTCTGACA | TCAGGATCGTGGTACTGAGCA |
| MDM2 | AGCCTGGCTCTGTGTGTAATA | CCTGATCCAACCAATCACCTGA |
| MMP1 | ACTCTGGAGTAATGTCACACCT | GTTGGTCCACCTTTCATCTTCA |
| MMP3 | CGGTTCCGCCTGTCTCAAG | CGCCAAAAGTGCCTGTCTTTA |
| MMP9 | ACCTCGAACTTTGACAGCGAC | GAGGAATGATCTAAGCCCAGC |
| MMP13 | TTTCAACGGACCCATACAGTTTG | CATGACGCGAACAATACGGTTA |
| Myc | CCACAGCAAACCTCCTCACAG | GCAGGATAGTCCTTCCGAGTG |
| P15 | GGTGCAGAGCTGTCGCTTTCAG | TCAGCCAGCCAGTTGGTTTCAC |
| P21 | CCTGTCACTGTCTTGTACCCT | CCCGCAGTATCTTGCCTCC |
| PPARβ/δ | AGGAGCCATTCTGTGTGTGA | TCCTGCCAGCAGAGAGTGAT |
| Smad3 | GAACGTCAACACCAAGTGCAT | ACGCAGACCTCGTCCTTCT |
| SOCS | GCTTTTTCGCCCTTAGCGTG | GAGGCAGTCGAAGCTCTCG |
| STAT3 | ATCACGCCTTCTACAGACTGC | CATCCTGGAGATTCTCTACCACT |
| STAT5b | TAAGAGGTCAGACCGTCGTGG | GCTGCCATGAACGATCACCA |
| TAK1 | GTTCCGTGTAAGGGCTTTGA | GAGCAGCTGCCACTTACCTTTACA |
| TGFβR2 | CGTGGAGTCGTTCAAGCAGACG | CCGCACCTTGGAACCAAATGG |
| VHL | GAGTCCGGCCCGGAAGAGTCCG | CATCGTGTGTCCCTGCATCTCT |

**Supplementary Table S2.** Percentage of apoptotic cells after exposure to indicated concentrations of MNNG and H_2_O_2_.

Apoptotic cells were determined by FACS analysis (10,000 events). Sum of Annexin V_high_/PI_low_ (early apoptosis) and Annexin V_high_/PI_high_ (late apoptosis) were considered apoptotic. Values (bold) denote significant increased apoptotic cells (%) when compared with untreated keratinocytes. Values were means of three independent experiments. Data were analyzed using FlowJo.

**Supplementary Table S3.** Percentage of necrotic cells after exposure to indicated concentration of MNNG and H_2_O_2_.

Necrotic cells (Annexin V_low_/PI_high_) were determined by FACS analysis (10,000 events). Values (bold) denote significant increased necrotic cells (%) when compared to untreated keratinocytes. Values were means of three independent experiments. Data were analyzed using FlowJo.

[**References**](https://wizfolio.com/?style=1&ver=3&UserID=8631&StyleName=Cancer%20Research)

1. Lam CRI, Tan MJ, Tan SH, Tang MBY, Cheung PCF, Tan NS et al.. TAK1 regulates SCF expression to modulate PKBα activity that protects keratinocytes from ROS-induced apoptosis. Cell Death Differ 2011;18:1120-1129.

2. Chong HC, Tan MJ, Philippe V, Tan SH, Tan CK, Ku CW et al.. Regulation of epithelial-mesenchymal IL-1 signaling by PPARbeta/delta is essential for skin homeostasis and wound healing. J Cell Biol 2009;184:817-831.
